# Large language models for primary care ophthalmic education: a systematic review

**DOI:** 10.3389/fmed.2026.1810098

**Published:** 2026-03-31

**Authors:** Shuang Li, Xiaoyan Wang, Yaqi Chen, Meiwen Tian, Peijie Lin, Mingying Lai, Ligang Jiang

**Affiliations:** 1 Fuyong People's Hospital, Shenzhen, China; 2 Shenzhen Eye Hospital, Shenzhen Eye Medical Center, Southern Medical University, Shenzhen, China; 3 Department of Ophthalmology, Quzhou Affiliated Hospital of Wenzhou Medical University, Quzhou People’s Hospital, Quzhou, Zhejiang, China

**Keywords:** large language models, ophthalmology education, primary care ophthalmology, retrieval augmented generation, virtual patients

## Abstract

**Background:**

Primary care physicians (PCPs) are a critical first contact for eye-health screening, risk stratification, and referral, yet ophthalmology training and point-of-care support in primary care remain insufficient. Recent advances in generative artificial intelligence (generative AI), particularly large language models (LLMs), may help address these gaps through conversational, scenario-based learning and structured feedback. However, the educational effectiveness, reproducibility, and safety boundaries of LLM-enabled tools in primary care ophthalmology remain unclear.

**Methods:**

We conducted a systematic review of studies evaluating or applying LLMs in ophthalmic education, training, assessment, or primary care–relevant clinical support. PubMed, Web of Science Core Collection, and Scopus were searched from January 1, 2020 to December 31, 2025 using combined terms related to LLMs/generative AI, ophthalmology, and education or assessment. Citation chaining was also performed to reduce omission. Two reviewers independently screened records and extracted data.

**Results:**

The evidence base is dominated by vignette-based benchmarks, comparative scoring studies, and evaluations conducted in limited-sample or controlled settings; prospective real-world validation using learner transfer, clinical behavior change, workflow impact, or patient outcomes remains scarce. Across studies, LLMs can serve as “cognitive apprenticeship” partners by externalizing clinical reasoning and enabling repeated practice in key-feature extraction, differential diagnosis, risk stratification, and referral-threshold decisions. Applications include triage/reasoning drills, virtual patient interviewing, and support for structured referrals, documentation, and patient education, often strengthened by retrieval-augmented generation. Most studies benchmarked outputs against expert consensus or guidelines, but scoring rubrics and reference standards varied widely, limiting cross-study comparability. Some reports noted that adding clinical photographs could reduce accuracy, suggesting current multimodal models are better suited for history-based reasoning than fine-grained image interpretation. Limitations include heterogeneity, rapid model iteration, reproducibility challenges, multimodal instability, and safety risks such as hallucination, bias, and automation bias. Commonly recommended safeguards include retrieval grounding, source attribution, red-flag checklists, and human-in-the-loop review.

**Conclusion:**

With clearly defined task scopes and robust safeguards, LLMs may improve the accessibility and efficiency of primary care ophthalmic education, but should augment rather than replace expert judgment. Future work should prioritize pragmatic multicenter trials, mixed-method implementation studies, and standardized cross-lingual evaluations to define safe and effective implementation pathways.

## 1 Introduction

The escalating global burden of eye disease presents a formidable public health challenge, particularly concerning major blinding conditions such as cataracts, refractive errors, and glaucoma (1–5). As the population ages (6), the demand for ophthalmic services is surging. Consequently, primary care physicians (PCPs)—equipped with fundamental capabilities in disease identification, stratified management, and standardized referral—have emerged as a critical pivot for enhancing eye health equity and service accessibility (7). However, there is a longstanding and severe supply–demand mismatch in ophthalmic clinical education and practice support for primary care. On one hand, PCPs receive limited specialty training during their medical education, leading to a lack of confidence in initial screening and referral decision-making when managing complex eye diseases (8). On the other hand, primary care institutions generally face constraints such as limited equipment, a lack of expert supervision, and a scarcity of continuing education resources. Traditional offline, centralized teaching models struggle to meet the individualized, fragmented, and highly clinical context-dependent training needs of PCPs (9). This results in low efficiency in translating knowledge into practice and creates regional disparities in ophthalmic service capabilities (10, 11).

In recent years, research into generative artificial intelligence (AI), and specifically large language models (LLMs), has grown rapidly within the field of medical education (12–16), offering a new pathway to break this deadlock. Unlike traditional retrieval tools, LLMs can not only organize medical knowledge in a conversational format (17) but also assume the roles of on-demand mentors or virtual standardized patients (SPs) in primary care settings (18). By simulating real-world consultation scenarios, LLMs can assist PCPs in performing clinical reasoning for cases of varying difficulty, conducting structured history-taking training, and interpreting diagnostic and treatment pathways. This transforms fragmented knowledge into repeatable clinical reasoning exercises, thereby significantly shortening the learning curve and optimizing judgment regarding referral thresholds (19, 20). However, the application of LLMs in primary care scenarios is accompanied by risks and challenges. The accuracy of their outputs, their ability to identify key “red flag” signs, and the potential for hallucinations directly impact patient safety (21–23). Therefore, establishing a transparent, auditable safety evaluation framework and clarifying the boundaries of AI in primary care ophthalmic decision support are core prerequisites for the implementation of this technology.

In this review, the term ‘PCPs’ is used as an umbrella term to describe frontline non-ophthalmologist physicians who undertake first-contact assessment, preliminary risk stratification, and referral for eye-related complaints in community or general medical settings. Depending on the healthcare system and study context, this category may include general practitioners (GPs), family physicians, emergency physicians, and other frontline doctors with gatekeeping or first-contact responsibilities. These labels are not fully interchangeable across countries: family physicians and GPs are usually central to longitudinal primary care, whereas emergency physicians mainly provide acute first-line assessment but may also participate in ophthalmic triage. To improve conceptual clarity and international applicability, we use “PCPs” as the overarching term throughout the manuscript and retain narrower practitioner labels only when referring to specific study populations.

Despite existing reviews on AI in general medical education, there is a lack of systematic evidence synthesis specifically targeting PCPs in ophthalmology—a specialty highly dependent on visual recognition and precise decision-making. Accordingly, this review focuses on the PCP population to systematically analyze the application forms, actual effectiveness, and challenges of LLMs in ophthalmic clinical education and practice support. The aim is to provide a reference for constructing an efficient and safe human-AI collaborative hybrid training paradigm oriented toward primary care.

## 2 Search strategy and study selection workflow

A systematic search was conducted in PubMed (covering the MEDLINE core index), Web of Science Core Collection, and Scopus, spanning the period from January 1, 2020, to December 31, 2025. The search strategy relied on a combination of three key conceptual domains: large language models/generative artificial intelligence, ophthalmology-related topics, and educational training and assessment scenarios. Keywords included “large language model,” “LLM,” “generative AI,” “ChatGPT,” “foundation model,” “ophthalmology,” “primary care education,” “training,” and “virtual patient,” among others. These were expanded using Boolean operators and controlled vocabulary (subject headings). To minimize the risk of omission, we manually screened the reference lists of included studies and key reviews. Additionally, citation chaining was performed in Web of Science to identify supplementary relevant literature. After removing duplicates, the search results underwent a screening process, during which primary reasons for exclusion were documented to ensure process traceability.

Studies were included if they met one of the following criteria: (1) studies that utilized or evaluated large language models in ophthalmic education, training, or assessment scenarios, covering tasks such as virtual patient and consultation training, image-related Q&A teaching, and instructional workflow assistance; or (2) studies outside the field of ophthalmology that provided governance or methodological frameworks transferable to medical education or clinical text generation, offering direct explanatory value to the research questions of this review.

Exclusion criteria included: non-peer-reviewed literature (e.g., preprints and conference abstracts); studies not involving large language models (e.g., traditional natural language processing only or image-only models without language interaction); studies unrelated to educational objectives and lacking transferable frameworks; and duplicate datasets or reports.

**Figure 1:**
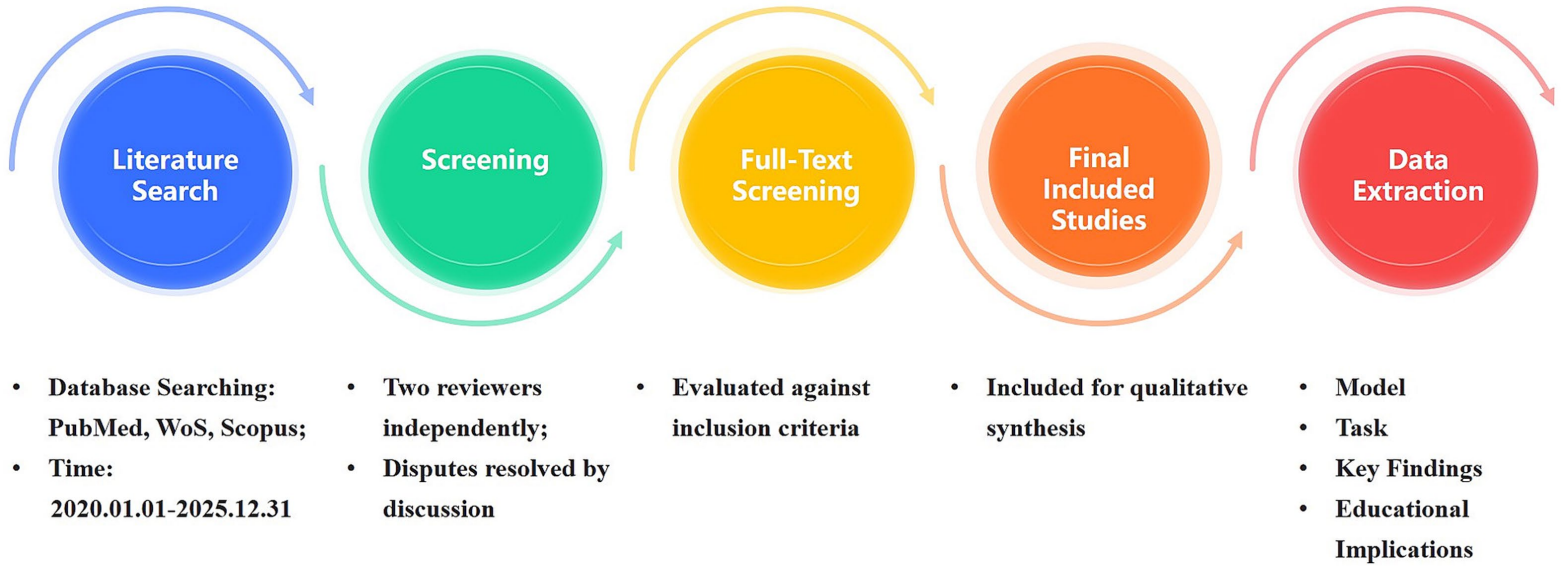
Literature search and study selection workflow.

**Figure 2:**
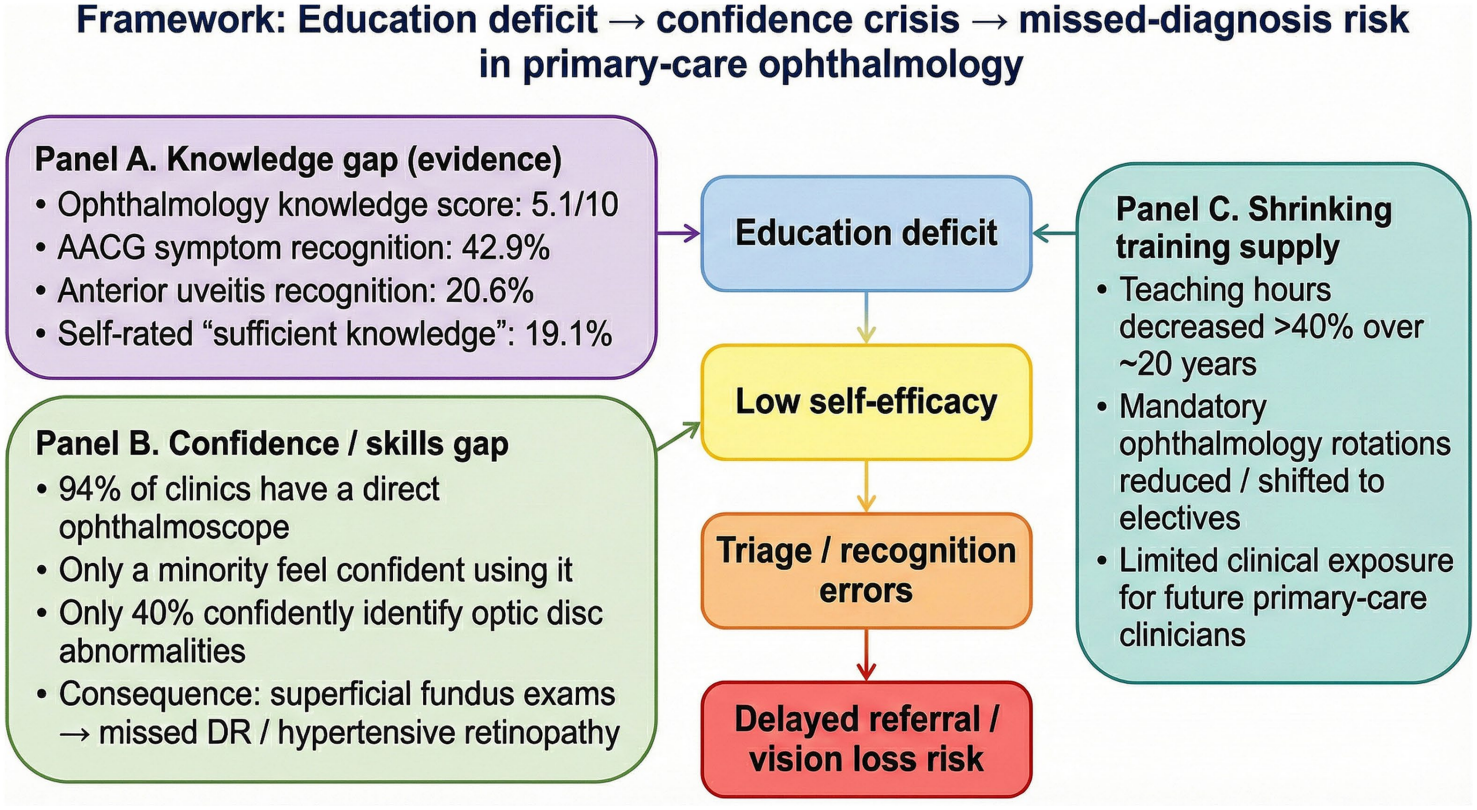
Conceptual framework linking education deficit to low self-efficacy and missed-diagnosis risk in primary care ophthalmology.

**Figure 3:**
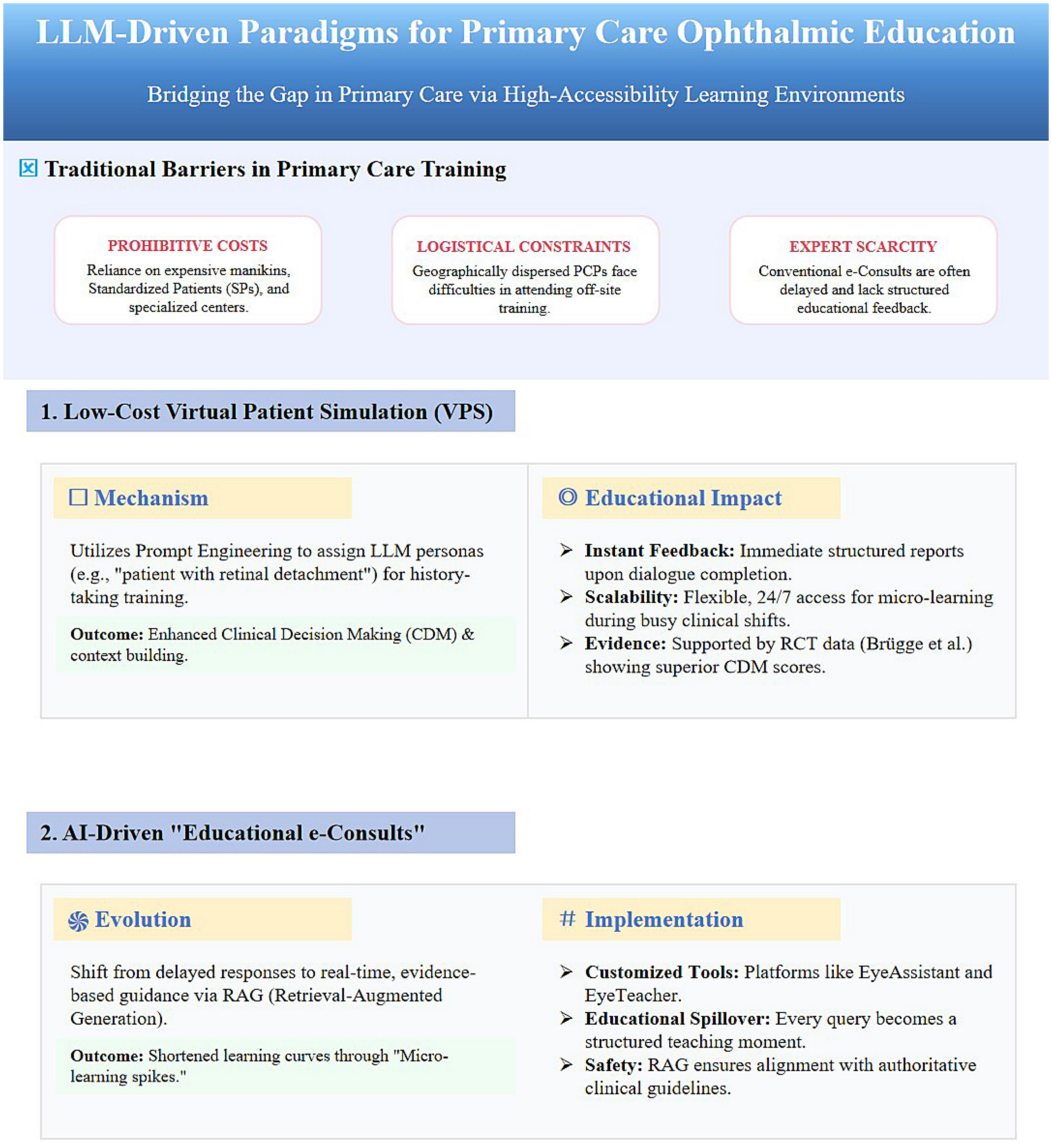
LLM-driven solutions for primary care ophthalmic education barriers.

Two authors independently screened titles and abstracts, followed by a full-text review to determine final inclusion. Disagreements were resolved through discussion or, if necessary, adjudicated by a third author. The methodological quality of the included studies was independently assessed by two researchers using established checklists appropriate to the study design, with discrepancies resolved through consensus (Figures 1–3).

## 3 Exploration of the application of LLMs in clinical education for PCPs

In the global health system, primary care settings are regarded as the first line of defense for eye health management, shouldering the critical responsibilities of disease prevention, early screening, triage of acute conditions, and long-term management of chronic eye diseases (24, 25). However, driven by accelerated population aging and the prevalence of metabolic diseases such as diabetes and hypertension (26, 27), the demand for ophthalmic care is witnessing exponential growth. In sharp contrast, the relative shortage and uneven distribution of ophthalmic specialists worldwide have intensified the “funnel effect” (28). As a result, a massive influx of patients with eye diseases floods into primary care, yet many PCPs—including GPs, family physicians, and emergency physicians—often lack sufficient specialized ophthalmic knowledge and clinical skills to address this challenge (29).

Large language models present an unprecedented technological opportunity to reconstruct the primary care ophthalmic education system and clinical support models. Distinct from traditional passive medical education—such as reading guidelines or attending lectures—LLMs, leveraging their vast medical knowledge bases, natural language understanding, and multi-turn conversational reasoning capabilities, can provide PCPs with just-in-time, interactive, and contextualized educational support and decision-making assistance (30).

### 3.1 Analysis of educational deficits and current clinical competence of PCPs

Prior to substantiating the application value of LLMs, it is imperative to conduct a comprehensive analysis—grounded in empirical data—of the knowledge deficits, scarcity of educational resources, and crisis of clinical confidence currently confronting PCPs. This serves not only as the logical premise for technological intervention but also as a baseline for evaluating the educational efficacy of LLMs. Multiple cross-national cross-sectional studies consistently indicate that PCPs possess a generally low level of ophthalmic knowledge and exhibit significant clinical blind spots. This paucity of knowledge is not confined to developing nations but is also prevalent in countries with relatively advanced healthcare systems, manifesting as a global, systemic educational deficiency. In a recent study by Othman et al. (31) targeting family physicians in Saudi Arabia, the average ophthalmic knowledge score was merely 5.1 out of 10, hovering on the borderline of adequacy. More critically, this score masks a severe insufficiency in the ability to identify vision-threatening conditions. Data reveal that only 42.9% of physicians could recognize the typical symptoms of acute angle-closure glaucoma, while the recognition rate for anterior uveitis was as low as 20.6%. This implies that in real-world clinical scenarios, over half of patients with acute glaucoma may be missed during the initial consultation, thereby missing the “golden window” for vision salvage and facing the risk of irreversible optic nerve damage. This phenomenon is corroborated in South America. A study in Brazil involving physicians in the Family Health Program (32) showed that only 19.1% of respondents believed they possessed sufficient ophthalmic knowledge for daily practice. Specifically regarding clinical procedures, only 56.5% knew how to correctly manage ocular perforation, and the proportion able to identify signs of acute glaucoma was only 44.3%. This lack of competency in managing ophthalmic emergencies directly leads to diagnostic delays and inappropriate interventions during the referral process. Furthermore, there is a substantial gap in the understanding of pediatric eye diseases; inexperienced residents demonstrate significant knowledge voids regarding the screening and referral of vision-threatening conditions in children, potentially resulting in permanent visual impairment (33).

Physician self-efficacy and clinical confidence are similarly low. A survey targeting GPs (34) found that although 94% of clinics were equipped with direct ophthalmoscopes, only a small fraction of physicians felt confident using the device, and merely 40% were confident in identifying optic disk abnormalities. This predicament—where equipment is available but underutilized and pathologies are present but unrecognized—renders fundus examination, a critical diagnostic tool, largely performative in primary care. Consequently, a vast number of patients with diabetic retinopathy (DR) or hypertensive fundus changes remain undiagnosed (35). The root cause of the weak ophthalmic competency among PCPs can be traced to the marginalization of ophthalmology curricula during medical education. A systematic review of global medical school ophthalmology education by Spencer et al. (8) revealed an alarming long-term trend: over the past two decades, the average duration of ophthalmology curricula in medical schools has been reduced by more than 40%. This educational decline is manifested not only in reduced class hours but also in the deprivation of clinical rotation opportunities. Many medical schools have removed ophthalmology from mandatory rotations, reclassifying it as an elective. Consequently, a large number of future PCPs never systematically encounter ophthalmic patients throughout their entire internship (36). The reduction in educational investment stands in sharp conflict with the explosive growth of medical knowledge. As medical curricula become increasingly crowded, ophthalmology—often viewed as a minor specialty—is frequently the first to be compressed. However, as the visual system serves as a crucial window into systemic health, the decline in its educational weight not only impacts the diagnosis and treatment of eye diseases but also weakens physicians’ capacity for the comprehensive assessment of systemic diseases.

### 3.2 Mechanisms and efficacy of LLM-assisted triage education in primary ophthalmology

Triage is one of the most core and high-frequency tasks in primary care and emergency ophthalmology settings. Physicians must perform risk stratification within a limited timeframe based on chief complaints, medical history, and preliminary examinations, subsequently deciding whether a patient requires immediate referral, scheduled referral, or management within primary care. This process is highly dependent on clinical reasoning capabilities (37). Current evidence regarding the use of LLMs for triage and reasoning training primarily stems from three types of studies: scenario simulations/benchmarking, scoring studies compared against experts, and a limited number of intervention studies. Since different evidence types support real-world generalizability to varying degrees, their efficacy must be interpreted within the framework of task boundaries and supervision guardrails (38).

First, LLMs can provide PCPs with low-cost, high-density simulation training opportunities by generating diverse clinical vignettes. This compensates for the issue where real-world caseloads in primary care are skewed toward common diseases, resulting in insufficient exposure to acute and critical conditions. Lyons et al. (39) evaluated the triage performance of ChatGPT-4 across 44 typical ophthalmic clinical scenarios; the results showed that the model included the correct diagnosis in its top three differentials in 93% of cases and judged triage urgency appropriately in 98%. Its overall performance surpassed that of some traditional symptom checkers and approached the level of trained ophthalmology residents on scenario-based tasks. These results suggest that LLMs can be utilized to enhance the frequency and feedback quality of “differential diagnosis–risk stratification–referral decision” exercises. However, it must be noted that scenario-based benchmarking is not equivalent to real-world performance; results are susceptible to prompt variations, the composition of evaluation datasets, and reference standards, presenting challenges regarding time sensitivity and reproducibility.

Second, from the perspective of educational psychology, the value of LLMs lies not only in providing conclusions but also in their ability—under prompt constraints—to output a structured Chain of Thought (CoT), thereby fostering transferable reasoning frameworks for learners (40). When PCPs input vague complaints, LLMs can be instructed to simulate expert reasoning flows, such as: (1) Extracting Key Features: Identifying critical search terms and “red flag” signs; (2) Constructing Differential Diagnoses: Listing possibilities such as acute angle-closure glaucoma, optic neuritis, or corneal ulcers; (3) Exclusionary Reasoning: Prompting further inquiry into symptoms like headache, nausea (pointing to glaucoma), or pain on eye movement (pointing to optic neuritis); (4) Risk Stratification and Management Recommendations: Clarifying whether the condition is an ophthalmic emergency and recommending referral timeframes. This immediate, explanatory feedback helps physicians gradually refine their illness scripts through repeated practice and more clearly distinguish common complaints that appear similar but carry different risk levels (e.g., red eye) (41). However, it is crucial to emphasize that explanatory reasoning output by LLMs is not inherently equivalent to correct reasoning. In educational applications, this should be combined with traceable evidence sources—such as Retrieval-Augmented Generation (RAG) sourcing, citations of guidelines/consensus, checklists, and instructor/peer review mechanisms—to prevent learners from internalizing rationalized errors.

Third, in high-pressure scenarios such as emergency departments and night shifts, LLMs can provide a degree of Clinical Decision Support (CDS) in controlled tasks. However, their role should be positioned as a “second opinion” or a source of structured prompts rather than a substitute for specialist decision-making. A prospective study by Kreso et al. (42) compared LLMs, including ChatGPT-4 and ChatGPT-4o, against human experts in emergency ophthalmic decision-making, suggesting that ChatGPT-4 achieved scores approaching those of human ophthalmologists in generating diagnostic and treatment plans. Addressing complex situations like ocular trauma, Xue et al. (43) evaluated the efficacy of multimodal ChatGPT in assisting emergency physicians with triage and surgical decisions. The results showed that when only textual medical record information was input, ChatGPT-4 Turbo achieved a diagnostic accuracy of 94.23–98.08%, indicating advantages in organizing medical history, summarizing injury mechanisms, and suggesting examinations. However, when ocular photographs were introduced, diagnostic accuracy dropped to 63.46%, reflecting that general-purpose multimodal models remain unstable in recognizing subtle signs and maintaining image consistency. Consistent with this, Antaki et al. (44) reported low detection performance and a certain degree of hallucination risk in general vision-language models applied to more granular Optical Coherence Tomography (OCT) tasks, indicating they do not yet meet clinical standards. Based on this evidence, the currently feasible path in primary and emergency care education is to prioritize text-based reasoning training and procedural risk stratification; image interpretation teaching and diagnostic support are better suited to integrated architectures combining specialized vision models and LLMs to enhance reliability and explainability. Advanced deep learning frameworks, such as the Multi-rater Prism, have demonstrated significant potential in handling inter-rater variability and improving segmentation calibration, providing a robust visual foundation that can complement the reasoning capabilities of LLMs (45). For example, deep learning-based systems have achieved high accuracy in diagnosing anterior segment diseases like pterygium, providing a reliable reference for primary screening (46). Similarly, the Meta-Eye foundation model (Meta-EyeFM) proposed by Soh et al. (47) and the evaluation of ophthalmic-specific foundation models (such as RETFound) by Kuo et al. (48) suggest that, compared to general-purpose models, foundation models pre-trained on specific medical data possess higher label efficiency and diagnostic potential in tasks like OCT, providing further evidence for general-specialist multimodal architectures.

Beyond direct clinical recommendations, LLMs possess implicit educational value in improving the quality of referral documentation. Referral letters written by PCPs are often information-poor, making it difficult for specialists to pre-assess the patient’s condition. Huang et al. (49) compared the performance of five LLMs, including ChatGPT-4o and DeepSeek-V3, in answering ophthalmic inquiries, finding that ChatGPT-4o and DeepSeek-V3 excelled in accuracy and logical coherence. Meanwhile, Wang et al. (50) further confirmed that through specific enhancement strategies (such as prompt engineering optimization), the safety of LLMs in medical Q&A was improved, effectively reducing the risk of misleading information. These evidences indicate that high-level LLMs can serve as a foundation for reliable knowledge sources. Transferred to physician education scenarios, LLMs can assist PCPs in transforming chaotic clinical notes into structured, professional referral letters. A study on LLMs detecting errors in ophthalmic medical records showed that ChatGPT-4o could identify clinical logical errors in records and propose corrections, effectively acting as an AI mentor for medical record quality control (Table 1).

**Table 1:** Summary of key studies on LLM-assisted triage in primary care and emergency ophthalmology settings.

Research source	Model version	Application scenario	Key results	Educational implications
Lyons et al. (39)	ChatGPT-4	Triage of 44 general ophthalmology clinical scenarios	Top-3 correct diagnosis rate 93%; Appropriateness of triage urgency judgment 98%	Suitable for high-frequency scenario simulation and training on “differential diagnosis — risk stratification — referral thresholds”
Kreso et al. (42)	ChatGPT-4, Llama-3	73 real-world emergency ophthalmology cases	No significant difference compared to human expert ratings	Can serve as an emergency “second opinion/structured prompt,” but requires final decision by humans
Xue et al. (43)	ChatGPT-4 Turbo	Diagnosis and surgical decision-making for ocular trauma cases	Text input accuracy 94.23–98.08%; Dropped to 63.46% after adding eye photographs	Strong textual logic, unstable image recognition; Suggests “prioritize text reasoning, specialized models needed for images”

### 3.3 Intelligent education and interdisciplinary collaboration in primary care chronic eye disease management

As DR, age-related macular degeneration (AMD), and glaucoma have become the leading causes of blindness (6), PCPs play a pivotal role in the long-term management of chronic eye diseases, the improvement of screening compliance, and patient education. This educational role is increasingly important because the quality of publicly accessible short-video education on chronic eye diseases such as diabetic retinopathy and AMD is variable, highlighting the need for more standardized and clinically grounded communication tools (51, 52). The application of LLMs is transforming the traditional “screening-referral” model into an integrated “screening-education-management” intelligent model. Diabetic fundus screening is a globally recognized challenge in blindness prevention. Although tele-retinal screening has been proven to effectively increase screening coverage (53–55), in many such programs, PCPs are responsible only for image acquisition and uploading. They often lack the ability to interpret results or explain complex fundus lesions to patients, as interpretation and grading are typically performed by remote reading centers, trained graders, or algorithms (56, 57). The DeepDR-LLM system developed and validated by Li et al. (58) offers a representative paradigm for this approach: a deep learning module automatically analyzes fundus photographs to identify features such as hemorrhages and exudates, followed by an LLM module that combines patient metabolic information (e.g., HbA1c, blood pressure) to generate empathetic, actionable, and personalized management advice in lay language. Related studies suggest that deploying such systems in primary care settings may improve self-management behaviors in newly diagnosed diabetic patients and enhance compliance with DR referrals. For PCPs, this standardized output of “image interpretation–risk explanation–management planning” also serves as contextualized learning material, helping them understand the clinical link between DR grading and systemic metabolic control in practice, thereby improving their capabilities in chronic disease follow-up and patient education. Notably, addressing regions with extreme resource scarcity lacking fundus imaging equipment, Choi et al. (59) proposed a more universally applicable, low-threshold solution: utilizing ChatGPT-4 to construct a risk calculator based on clinical text data (e.g., disease duration, blood glucose levels), enabling preliminary DR risk stratification without reliance on imaging. Similarly, Gong et al. (60) confirmed the effectiveness of nomograms based on non-imaging clinical data in predicting the risk of vision-threatening diabetic retinopathy, further validating the feasibility of using electronic health records for risk stratification in primary care settings. This model emphasizes accessibility and rapid deployment, offering an alternative viable path for chronic disease management at the primary level; however, its applicable boundaries and clinical safety require further verification in larger samples and real-world scenarios.

One of the critical challenges PCPs face is the need to simultaneously address patients’ multiple comorbidities and long-term risk management requirements within a limited timeframe (61). Ophthalmology is not an isolated discipline; systemic factors such as diabetes, hypertension, and kidney disease can all influence the onset and progression of retinopathy. LLMs possess potential advantages in integrating interdisciplinary information and generating structured recommendations. Existing studies evaluating the performance of LLMs on DR screening advice in real-world case scenarios suggest that, after synthesizing multiple clinical variables, LLMs can provide personalized recommendations for initial screening windows or screening intervals that align with guideline logic. This offers decision support for risk stratification and follow-up planning in primary care settings (62). Furthermore, systemic risk factors (particularly blood pressure control) are closely related to the risk of diabetic microvascular complications. Relevant randomized controlled trials suggest that intensive blood pressure management can reduce the risk of microvascular events and may influence the onset and progression of DR (63). In this context, LLMs hold promise for translating complex pathophysiological mechanisms and risk management logic into health education language that is understandable and actionable for patients. This enhances the quality and efficiency of doctor-patient communication in chronic disease management and promotes the implementation of holistic care and systemic thinking in primary care practice (Table 2).

**Table 2:** Key evidence for LLMs in primary care chronic eye disease management and interdisciplinary collaboration.

Research source	Study scheme	Technical path	Application scenario	Key conclusions
Wong et al. (58)	DeepDR-LLM	Interpreting fundus images and LLMs integrating metabolic data to generate recommendations	Primary care DR screening and follow-up management	May improve self-management behaviors and increase DR referral compliance; outputs can serve as contextualized learning materials
Choi et al. (59)	Text-based Risk Calculator	Constructing risk stratification tools based solely on clinical text/indicators	Initial DR risk screening in areas without imaging equipment	Low threshold, high accessibility; can be used for preliminary risk stratification
Gopalakrishnan et al. (62)	Evaluation of screening recommendations in multivariate scenarios	LLMs integrate comorbidities and risk factors to generate screening interval recommendations	Complex scenarios such as pregnancy with diabetes/family history/stages of kidney disease	Can generate individualized screening schedule recommendations consistent with guideline logic

### 3.4 A new paradigm for simulation teaching and interactive learning in primary ophthalmology

Traditional simulation-based education relies heavily on costly high-fidelity mannequins, SPs, and specialized training centers—resources that remain an unattainable luxury for PCPs dispersed across community and rural settings. Wang et al. (64) pointed out that for low- and middle-income countries (LMICs), tools like ChatGPT, due to their minimal deployment costs and multilingual capabilities, hold the promise of bridging the infrastructure gap and serving as a critical lever to address shortages in medical education resources. Leveraging these generative capabilities, LLMs have paved a new path for constructing low-cost, highly accessible, and 24/7 virtual teaching environments (65). A randomized controlled trial (RCT) by Brügge et al. (19) provides robust evidence for the application of LLMs in simulation-based education. In this study, LLMs were utilized to simulate virtual patients with specific symptoms, allowing medical students to practice history taking. Results indicated that the experimental group, which received LLM-based simulation training with structured feedback, scored significantly higher in Clinical Decision Making (CDM) than the control group, with marked improvements in sub-dimensions such as context construction and information gathering. This model is fully transferable to primary care ophthalmic education. Through simple prompt engineering, ChatGPT can be configured to act as a patient with retinal detachment, enabling PCPs to practice consultation skills. Upon completion of the dialog, the LLM can immediately generate an evaluation report. A systematic review by Li et al. (66) supports this view, noting that LLM-based virtual patient systems offer personalized, scalable, and location-independent learning experiences, making them highly suitable for the fragmented learning schedules of practicing physicians.

Electronic consultations (e-Consults) represent a vital mechanism for PCPs to seek non-face-to-face advice from specialists, carrying significant educational benefits. However, traditional e-Consults are constrained by the time and energy of specialists, often resulting in delayed and brief responses (67). Customized generative pre-trained transformer (GPT) tools such as EyeAssistant and EyeTeacher, developed by Sevgi et al. (68), demonstrate how LLMs can utilize RAG technology to answer clinical questions based on authoritative clinical guidelines. Such AI-driven e-Consults can provide immediate responses to PCP inquiries. This not only resolves clinical uncertainties but also transforms every consultation into a micro-learning opportunity, gradually enhancing the knowledge reserve of PCPs through high-frequency reinforcement (Table 3).

**Table 3:** Key evidence for LLM-driven simulation teaching and interactive learning.

Research source	Design type	Key conclusions	Ways to transfer to primary ophthalmology
Wang et al. (64)	Perspective/Evidence review	In LMICs, low-cost and multilingual capabilities like ChatGPT can bridge the infrastructure gap	Supports feasibility arguments for deploying “pocket tutors/virtual patients” at the primary level
Brügge et al. (19)	RCT	LLMs virtual patients and structured feedback can improve CDM scores	Can use ophthalmology chief complaints and emergency scripts to build virtual case training
Li et al. (66)	Systematic review	LLMs virtual patient systems have advantages of personalization, scalability, and being unlimited by time and space	Suitable for fragmented learning and continuing education for in-service physicians
Sevgi et al. (68)	Custom GPT + RAG (EyeAssistant/EyeTeacher)	Uses authoritative guidelines to answer clinical questions in real-time and transforms consultations into micro-learning	Can serve as an alternative/supplementary path for AI-driven e-Consult and guideline learning

## 4 Challenges and limitations

While this review highlights the potential of LLMs in primary care ophthalmic education and practice support, these findings must be interpreted with caution. First, existing evidence is primarily derived from benchmarking studies, case-scenario simulations, limited-sample observational studies, and controlled educational evaluations. Real-world prospective validation remains insufficient, and high-quality research using endpoints such as learning transfer, clinical behavior change, workflow integration, and patient outcomes is still scarce. Second, significant heterogeneity exists across studies regarding prompt design, task boundaries, evaluation dataset composition, reference standards, and scoring scales. Coupled with the rapid iteration of model versions, this renders conclusions time-sensitive and poses challenges to reproducibility. Third, included studies suggest that general-purpose multimodal models remain unstable in recognizing subtle signs in ophthalmic imaging, with performance declining in some tasks when photographs are introduced. At this stage, LLMs are more suitable for history taking, clinical reasoning training, procedural risk stratification, documentation structuring, and patient communication support, rather than replacing instruction on slit-lamp examination or fine-grained image interpretation (69). Fourth, safety risks such as hallucinations, bias, and automation bias require continuous evaluation and governance within frameworks of traceable evidence chains (e.g., RAG sourcing), clinical guardrails, human oversight, and standardized reporting. Importantly, for primary care physicians without formal AI training, automation bias should be regarded not only as a theoretical concern but also as a practical implementation challenge. A feasible mitigation pathway is to embed LLM use within a structured human-AI collaborative framework, including targeted AI literacy training, explicit instruction on recognizing unsupported recommendations, referral-threshold and red-flag checklists, transparent uncertainty disclosure, and mandatory human verification in high-risk scenarios. Future research should move beyond general calls for more validation and instead adopt concrete, implementation-oriented designs. Examples include multicenter comparative studies or cluster-randomized trials evaluating whether LLM-based virtual triage training improves PCP referral accuracy in acute red-eye or sudden-vision-loss scenarios compared with conventional online education; mixed-method studies examining clinician trust, usage patterns, cognitive dependence, and workflow barriers in LLM-assisted diabetic retinopathy counseling or chronic eye-disease follow-up; and cross-lingual standardized benchmarking studies assessing whether safety, reasoning quality, and educational value remain stable across languages, healthcare systems, and resource settings. Such work will be essential to define optimal human-AI collaboration boundaries and feasible educational implementation pathways (70).

## 5 Conclusion

This systematic review suggests that primary care ophthalmic education and practice support are undergoing a structural shift driven by generative AI. Within clear task boundaries and constraints, LLMs have evolved from simple “information retrieval–answer output” tools into “cognitive apprenticeship partners” capable of externalizing clinical reasoning, simulating contextual dialogs, and providing immediate feedback (71, 72). This is particularly critical for primary care settings, where the core challenge is not a lack of clinical guidelines, but rather a deficit in high-frequency training and timely corrective opportunities to rapidly map knowledge to specific patient complaints and contexts. Existing evidence indicates that LLMs have substantial potential in virtual patient consultation, initial screening, triage, referral decision-making exercises, and structured patient-education support. Specifically, under the constraints of structured prompts, they facilitate the formation of reasoning frameworks—encompassing “key feature extraction–differential diagnosis–risk stratification–management thresholds”—thereby increasing practice density and optimizing learning curves (39–41). In chronic disease management pathways, LLMs are poised to act as “amplifiers” of explanation and communication. Taking DR as an example, the integrated “Deep Learning + LLM” paradigm can transform technical grading and lesion information into understandable, actionable, and empathetic education and follow-up advice. This assists PCPs in extending a single screening event into a trigger for structured health education and long-term management, promoting the formation of a “screening–education–management” closed loop.

Simultaneously, this review emphasizes that the usability of LLMs is highly dependent on task type and the robustness of guardrails. Current research remains dominated by benchmarking and scenario simulations; real-world evidence is limited, and reproducibility is compromised by rapid model iteration. Furthermore, multimodal models remain unstable in recognizing subtle signs in ophthalmic imaging. This suggests that the more prudent path at present is to prioritize text-based reasoning, history-based clinical training, documentation support, and procedural risk stratification, while proceeding cautiously with image interpretation teaching (69). Therefore, implementation should follow a governance framework based on stratified access, auditable outputs, and mandatory human oversight. In practical terms, this requires integrating prompt engineering, evidence retrieval, and AI critical appraisal into competency training (73); utilizing RAG sourcing, red-flag and referral-threshold checklists, transparent uncertainty disclosure, and structured human review to reduce the risks of hallucination and automation bias; and ensuring that final decision-making authority remains with human clinicians in high-risk scenarios (74, 75). For PCPs without formal AI training, such safeguards are especially important to prevent over-reliance on fluent but unsupported outputs and to support safer human-AI collaboration in real-world educational and triage settings.

Overall, LLMs are better suited as educational enhancers designed to improve accessibility and efficiency under strict governance and assessment frameworks, rather than as substitutes for traditional mentors. If supported by robust safety guardrails, prospective real-world validation, and implementation-oriented evaluation, LLMs hold the promise of alleviating the pressures on primary care ophthalmic education and workforce supply, thereby aiding the advancement of universal eye health goals.
